# GC-biased gene conversion in X-chromosome palindromes conserved in human, chimpanzee, and rhesus macaque

**DOI:** 10.1093/g3journal/jkab224

**Published:** 2021-07-08

**Authors:** Emily K Jackson, Daniel W Bellott, Helen Skaletsky, David C Page

**Affiliations:** 1 Whitehead Institute, Cambridge, MA 02142, USA; 2 Howard Hughes Medical Institute, Whitehead Institute, Cambridge, MA 02142, USA; 3 Department of Biology, Massachusetts Institute of Technology, Cambridge, MA 02139, USA

**Keywords:** *Key words*: X chromosome, palindrome, primate, comparative genomics, GC-biased gene conversion, evolution

## Abstract

Gene conversion is GC-biased across a wide range of taxa. Large palindromes on mammalian sex chromosomes undergo frequent gene conversion that maintains arm-to-arm sequence identity greater than 99%, which may increase their susceptibility to the effects of GC-biased gene conversion. Here, we demonstrate a striking history of GC-biased gene conversion in 12 palindromes conserved on the X chromosomes of human, chimpanzee, and rhesus macaque. Primate X-chromosome palindrome arms have significantly higher GC content than flanking single-copy sequences. Nucleotide replacements that occurred in human and chimpanzee palindrome arms over the past 7 million years are one-and-a-half times as GC-rich as the ancestral bases they replaced. Using simulations, we show that our observed pattern of nucleotide replacements is consistent with GC-biased gene conversion with a magnitude of 70%, similar to previously reported values based on analyses of human meioses. However, GC-biased gene conversion since the divergence of human and rhesus macaque explains only a fraction of the observed difference in GC content between palindrome arms and flanking sequence, suggesting that palindromes are older than 29 million years and/or had elevated GC content at the time of their formation. This work supports a greater than 2:1 preference for GC bases over AT bases during gene conversion and demonstrates that the evolution and composition of mammalian sex chromosome palindromes is strongly influenced by GC-biased gene conversion.

## Teaser

Gene conversion is GC-biased. In this issue, Jackson et al. leverage the high gene conversion rates that maintain large palindromes on the primate X chromosome to estimate the magnitude of GC bias over the past 25 million years, finding a 2:1 preference for GC bases over AT bases. Using this unconventional approach, the current study corroborates earlier estimates of the magnitude of GC bias derived from analyses of human meioses, and partially explains elevated GC content in X palindromes.

## Introduction

Homologous recombination maintains genome integrity through the repair of double-stranded DNA breaks, while also promoting genetic innovation through programmed reshuffling during meiosis. Homologous recombination can produce crossover events, in which genetic material is exchanged between two DNA molecules, or non-crossover events. Crossover events and non-crossover events both result in gene conversion, the non-reciprocal transfer of DNA sequence from one homologous template to another. When the templates involved in gene conversion are not identical, gene conversion can be biased, resulting in the preferential transmission of one allele over another (reviewed in Galtier *et al.* 2001; Marais 2003; Duret and Galtier 2009). In particular, GC alleles are generally favored over AT alleles, leading to a strong correlation between GC content and recombination rates across the genome. GC-biased gene conversion is widespread across taxa, including plants (Muyle *et al.* 2011), yeast (Mancera *et al.* 2008), birds (Smeds *et al.* 2016), rodents (Montoya-Burgos *et al.* 2003; Clément and Arndt 2011), humans (Odenthal-Hesse *et al.* 2014; Williams *et al.* 2015; Halldorsson *et al.* 2016), and other primates (Galtier *et al.* 2009; Borges *et al.* 2019).

While early evidence for GC-biased gene conversion was indirect (Galtier *et al.* 2001; Marais 2003), two recent studies identified gene conversion events in humans directly using three-generation pedigrees (Williams *et al.* 2015; Halldorsson *et al.* 2016). This approach enabled calculation of the magnitude of GC bias, defined as the frequency at which gene conversion at a locus containing one GC allele and one AT allele results in transmission of the GC allele. Williams *et al.* (2015) identified 98 autosomal non-crossover gene conversion events at loci with one GC allele and one AT allele, and found that 63 (68%) transmitted the GC allele. Halldorsson *et al.* (2016) analyzed autosomal crossover and non-crossover gene conversion events separately, and found GC biases of 70.1% and 67.6%, respectively. The magnitude of GC bias may vary across different genomic positions: Another study used sperm typing to examine allele transmission at six autosomal recombination hotspots, and found evidence for GC-biased transmission at two hotspots, but unbiased transmission at the other four hotspots (Odenthal-Hesse *et al.* 2014).

Mammalian sex chromosomes contain large, highly identical palindromes, with arms that can exceed 1 Mb in length and arm-to-arm identities greater than 99% (Skaletsky *et al.* 2003; Warburton *et al.* 2004; Hughes *et al.* 2010, 2012; Mueller *et al.* 2013; Soh *et al.* 2014; Hughes *et al.* 2020; Jackson *et al.* 2021). Near-perfect identity between palindrome arms is maintained by high rates of ongoing gene conversion (Rozen *et al.* 2003), which may make palindromes uniquely susceptible to the effects of GC-biased gene conversion (Hallast *et al.* 2013; Skov *et al.* 2017). Recently, we generated high-quality reference sequence for 12 large palindromes that are conserved on the X chromosomes of human, chimpanzee, and rhesus macaque, demonstrating a common origin at least 25 million years ago (Jackson *et al.* 2021). Here, we use a comparative genomic approach combined with evolutionary simulations to analyze the impact and magnitude of GC-biased gene conversion in primate X-chromosome palindromes. We find that GC content is elevated in palindrome arms relative to flanking sequence, and that recent nucleotide replacements in human and chimpanzee palindrome arms are approximately one-and-a-half times as GC-rich as the ancestral bases that they replace. Using simulations of palindrome evolution, we show that our observed pattern of nucleotide replacements is consistent with a magnitude of GC bias of about 70%, which supports recent estimates derived from analyses of human meioses using an orthogonal approach (Williams *et al.* 2015; Halldorsson *et al.* 2016).

## Materials and methods

### Human mutation rate

Three recent publications used whole-genome shotgun sequencing data from related individuals to calculate human mutation rates of around 1.2 × 10^−8^ mutations per nucleotide per generation (Roach *et al.* 2010; Kong *et al.* 2012; Jónsson *et al.* 2017). However, these publications used only autosomal data, while the human X chromosome may have a lower mutation rate than autosomes due to its unique mode of transmission (Schaffner 2004). To our knowledge, similarly high-quality estimates of the human X-chromosome mutation rate do not exist. To estimate the mutation rate for the human X chromosome, we examined Supplementary Table S4 from Jónsson *et al.* (2017), which provides information for all autosomal and X-chromosome mutations detected in their dataset. Supplementary Table S4 reports 2694 X-chromosome mutations from 871 probands, or around 3.1 mutations per generation. To calculate the autosomal mutation rate, Jónsson *et al.* (2017) divided the number of autosomal mutations per generation by the number of autosomal base pairs with adequate coverage depth in their dataset. We therefore divided 3.1 X-chromosome mutations per generation by the length of the X chromosome in hg38 (156,040,895 base pairs) multiplied by the fraction of autosomal base pairs with adequate coverage (93.3%), which we assume here is similar to the fraction of X-chromosome base pairs with adequate coverage. This approach yielded an estimated human X-chromosome mutation rate of 1.06 × 10^−8^ mutations per nucleotide per generation. This value is about 20% lower than the value calculated by Jónsson *et al.* (2017) for autosomes (1.28 × 10^−8^ mutations per nucleotide per generation), consistent with predictions that mutation rates are lower on X chromosomes than on autosomes.

### GC content of primate X-chromosome palindromes

We calculated the GC content for each palindrome (Arm 1, spacer, and flanking sequence) using custom Python code. We performed all analyses using clones sequenced by Jackson *et al.* (2021). For flanking sequence, we used available sequence upstream and downstream of palindrome arms that was present in all three species. For example, if the human clones for a given palindrome contained 3′ sequence that was not sequenced in chimpanzee and rhesus macaque, we trimmed the human sequence to contain only the portion alignable between all three species. Visualizations were generated using ggplot2 in R (Wickham 2016; R Core Team 2020).

### Generation of sequence alignments

Sequence alignments were performed using ClustalW with default parameters (Thompson *et al.* 1994). To identify and exclude regions of poor alignment, ClustalW sequence alignments were scanned using a sliding 100-bp window and filtered to exclude windows with fewer than 60 matches between species, using custom Python code (Jackson *et al.* 2021).

### Calculation of divergence

Divergence was calculated by generating pairwise alignments using ClustalW, then calculating p-distance with MEGA X (Kumar *et al.* 2018). For alignment of arms between species, we generated pairwise alignments using Arm 1 from each species (Jackson *et al.* 2021).

### Simulations

Our simulations were designed to model the evolution of a palindrome present in the common ancestor of human, chimpanzee, and rhesus macaque, and maintained in all three lineages until the present. For each iteration, we initialized an ancestral palindrome with each nucleotide chosen at random based on the median characteristics of conserved primate X-chromosome palindromes (arm length: 37 kb, arm-to-arm identity: 99.953%, GC content: 46%). Each ancestral palindrome then underwent rounds of substitution followed by intra-chromosomal gene conversion, with two branching events to account for the divergence of human, chimpanzee, and rhesus macaque (see below for the calculation of the number of generations in each branch). Simulation parameters included the substitution rate for each evolutionary branch, relative rates for different types of substitutions (*i.e.*, the neutral substitution matrix), and the frequency and GC bias of intra-chromosomal gene conversion, with parameter values selected as described below. Simulations were implemented with custom Python code.

### Estimation of generation numbers for simulations

Divergence times for human *vs* chimpanzee and for human *vs* rhesus macaque are estimated at about 7 and 29 million years, respectively (Kumar *et al.* 2017). Generation times for primates vary between species, with estimated generation times around 30 years for humans (Tremblay and Vézina 2000; Matsumura and Forster 2008), 25 years for chimpanzee (Langergraber *et al.* 2012), and 11 years for rhesus macaque (Gage 1998; Xue *et al.* 2016). For simplicity, we assumed an intermediate value of 20 years per generation for all branches. Using these values, we estimated a total of 1,450,000 generations for the branch from the common human–chimpanzee–rhesus macaque (HCR) ancestor to rhesus macaque (Branch 1), 1,100,000 generations for the branch from the common HCR ancestor to the common human–chimpanzee (HC) ancestor (Branch 2), and 350,000 generations each for the branches from the common HC ancestor to chimpanzee and to human (Branches 3 and 4, respectively). For a discussion of the impact of generation numbers on our simulations, see Supplementary Note S2.

### Estimation of substitution rates for simulations

Substitution rates per generation can be inferred from the nucleotide divergence observed between species of known divergence times. We calculated these rates for each branch of our simulated evolutionary tree as follows:

*Substitution rate: Human* *vs* *chimpanzee* (Jackson *et al.* 2021) Palindrome arm divergence: 0.84% (see above) Generations: 350,000 * 2 = 700,000 Substitution rate: 1.20 × 10^−8^ substitutions per base per generation. 

*Substitution rate: Human* *vs* *rhesus macaque* (Jackson *et al.* 2021)  Palindrome arm divergence: 5.4% (see above) Generations: 1,450,000 * 2 = 2,900,000  Substitution rate: 1.86 × 10^−8^ substitutions per base per generation. 

The human–chimpanzee substitution rate was mapped directly onto Branches 3 and 4. The human–rhesus macaque substitution rate was mapped directly onto Branch 1. For Branch 2, we calculated the substitution rate such that the expected divergence along Branch 1 would equal the expected divergence along Branch 2 + Branch 3: (Branch 2 rate * 1,100,000 generations) 2.7% = 0.42% + (Branch 2 rate * 1,100,000 generations)  Branch 2 rate: 2.07 × 10^−8^ substitutions per base per generation. 

Note that for the Branch 2 calculation we assume symmetry of divergence, *i.e.*, divergence between two lineages is divided equally between them.

To confirm that our substitution rates were reasonable, we converted our values to per-year substitution rates assuming a generation time of 20 years, and compared these rates to previously published values. All three of our per-year substitution rates fall within confidence intervals for the same species estimated using autosomal data (Scally and Durbin 2012). Our values fell near the lower end of the confidence intervals, consistent with the prediction that substitution rates on the X chromosome should be slightly lower than on autosomes. Note that our estimated substitution rates represent average rates of sequence evolution over millions of years, and thus differ from the present-day mutation rate reported above for the human X chromosome, which was calculated using data from a single generation (Jónsson *et al.* 2017). Single-generation mutation rates are known to differ from average substitution rates over long evolutionary timescales, likely due to a recent slowdown in the mutation rate in humans and great apes (Scally *et al.* 2012). For a discussion of the impact of substitution rates on our simulations, see Supplementary Note S2.

### Estimation of neutral substitution matrix for simulations

Neutral substitution patterns between species do not follow a uniform distribution: Transitions are more common than transversions, and substitutions that replace a strong base (GC) with a weak base (AT) are more common than substitutions in the opposite direction (Petrov and Hartl 1999; Zhang and Gerstein 2003; Duret and Arndt 2008). In addition to branch-specific substitution rates, we therefore also sought to determine a reasonable pattern of neutral substitutions for our simulations.

We identified neutral substitutions using alignments from 3.8 Mb of gene-masked sequence flanking X-chromosome palindromes, using parsimony to infer substitution events in human and chimpanzee with rhesus macaque as an outgroup. From this, we calculated seven different substitution rates (Table 1).

**Table 1:** Neutral substitution matrix

Substitution	Substitution rate (substitutions/nt/generation)
AT → TA	1.64 × 10^−^^9^
AT → CG	1.93 × 10^−^^9^
AT → GC	8.04 × 10^−^^9^
CG → GC	2.98 × 10^−^^9^
CG → AT	3.22 × 10^−^^9^
CG → TA (non-CpG)	1.02 × 10^−^^8^
CG → TA (CpG)	9.58 × 10^−^^8^

The overall neutral substitution rate (K) can be calculated as described in Duret and Arndt (2008):

$$K=\;F_{\mathrm{GC}}\left(R_{\mathrm{CG}\to \mathrm{GC}}+{R_{\mathrm{CG}\to \mathrm{AT}}+\;R}_{\mathrm{CG}\to \mathrm{TA}\left(\mathrm{non}-\mathrm{CpG}\right)}\right)+\;F_{\mathrm{AT}}\left(R_{\mathrm{AT}\to \mathrm{TA}}+R_{\mathrm{AT}\to \mathrm{CG}}+R_{\mathrm{AT}\to \mathrm{GC}}\right)+\;F_{\mathrm{CpG}}(R_{\mathrm{CG}\to \mathrm{TA}\left(\mathrm{CpG}\right)})$$

where F_GC_, F_AT_, and F_CpG_ represent the frequencies of each site and R_AA→BB_ represents the frequencies of each substitution. Using the substitution rates above combined with the observed frequencies of each site (F_GC_: 0.396, F_AT_: 0.596, F_CpG_: 0.08), we found that K = 1.42 × 10^−8^ substitutions per nucleotide per generation. We then combined the categories CG→TA (non-CpG) and CG→TA (CpG) into a single rate CG→TA as follows:

$$R_{\mathrm{CG}\to \mathrm{TA}}=\left[F_{\mathrm{GC}}\;\left(R_{\mathrm{CG}\to \mathrm{TA}\left(\mathrm{non}-\mathrm{CpG}\right)}\right)+F_{\mathrm{CpG}}\;\left(R_{\mathrm{CG}\to \mathrm{TA}\left(\mathrm{CpG}\right)}\right)\right]/\left(F_{\mathrm{GC}}+F_{\mathrm{CpG}}\right)=1.18\;\times \;10^{-8}$$

substitutions per nucleotide per generation

We do not expect combining rates for CpG and non-CpG substitutions to affect either of our simulation output metrics (Figures 3, B and C: Fraction GC derived-Fraction GC ancestral at sites of nucleotide replacements; Figure 3D: Fraction GC overall) because these metrics are agnostic to the context in which each fixed nucleotide replacement occurred.

The substitution rates above were calculated using substitutions in flanking sequence since the divergence of chimpanzee and human; however, each evolutionary branch in our simulation has a different overall substitution rate (see section above). For each branch, we therefore divided the substitution rates above by the original overall substitution rate of 1.42 × 10^−8^ substitutions per nucleotide per generation, then multiplied by the branch-specific overall substitution rate. This kept the relative ratios between different substitution types constant, while accounting for different overall substitution rates in each branch. The effects of reasonable alterations of this neutral substitution matrix, including adjusting for possible under-estimation of the CpG substitution rate due to artifacts of parsimony, are described in Supplementary Note S3.

### Calculation of GC*

We used parsimony to identify fixed substitutions in chimpanzee and human palindrome arms using rhesus macaque as an outgroup, as described above for flanking sequence. We then calculated GC* separately for palindrome arms and for flanking sequence using the following equation (Hershberg and Petrov 2010; Bolívar *et al.* 2016):

$$\frac{{µ}_{w\to s}}{{µ}_{w\to s}\;+\;{µ}_{s\to w}}$$

where $\mu_{w\to s}\;=\;R_{\mathrm{AT}\to \mathrm{CG}}+R_{\mathrm{AT}\to \mathrm{GC}},\;$and $\mu_{s\to w}=\;R_{\mathrm{CG}\to \mathrm{AT}\;}+R_{\mathrm{CG}\to \mathrm{TA}}$.

## Results

### High rates of intrachromosomal gene conversion in arms of primate X-chromosome palindromes

To understand the role of GC-biased gene conversion in the evolution of primate X-chromosome palindromes, we first calculated the rate of intrachromosomal gene conversion between palindrome arms. Sequence identity between palindrome arms depends on the balance between two evolutionary forces: The rate at which new mutations arise in each arm, and the rate at which gene conversion between arms homogenizes the resulting sequence differences. The rate of intrachromosomal gene conversion can therefore be calculated using the formula *c* = 2 μ/*d*, where μ represents the mutation rate, and *d* represents the fraction divergence between arms (Rozen *et al.* 2003). Among 12 X-chromosome palindromes conserved between human, chimpanzee, and rhesus macaque, we found a median divergence between arms of 4.7 × 10^−4^ differences per nucleotide, or around one difference per 2200 nucleotides. Assuming a mutation rate of 1.06 × 10^−8^ mutations per nucleotide per generation (Roach *et al.* 2010; Kong *et al.* 2012; Jónsson *et al.* 2017; see Materials and methods), we calculated a gene conversion rate of 4.5 × 10^−5^ events per nucleotide per generation for primate X-chromosome palindromes. This value is nearly eight times the recent estimate of 5.9 × 10^−6^ gene conversion events per nucleotide per generation across human autosomes (Williams *et al.* 2015; Halldorsson *et al.* 2016), highlighting the rapid pace of genetic exchange between sex chromosome palindrome arms.

### GC content is elevated in primate X-chromosome palindrome arms compared to flanking sequence

**Figure 1:**
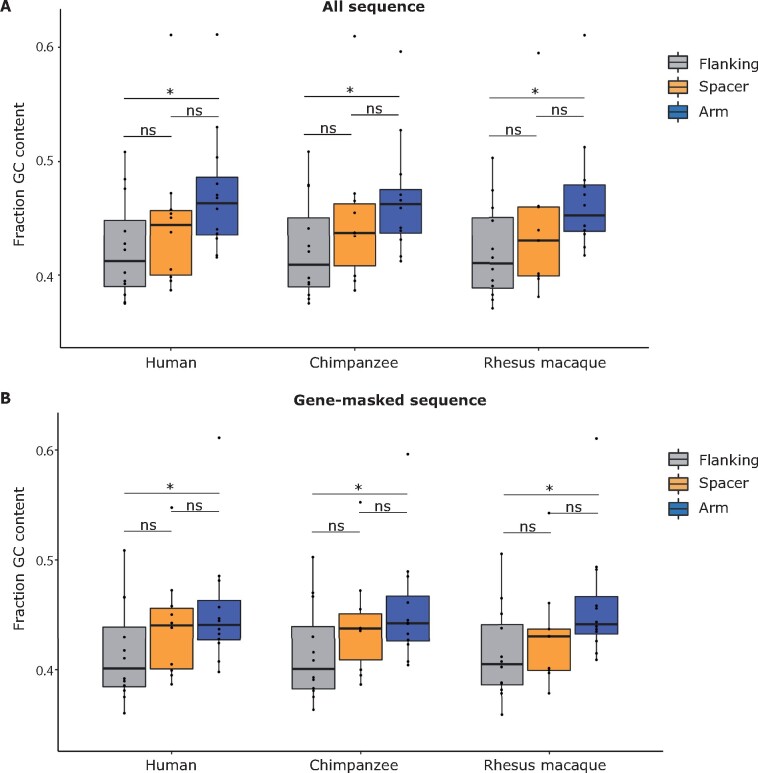
GC content is elevated in primate X-chromosome palindrome arms compared to flanking sequence. GC content measured in 12 palindromes conserved between human, chimpanzee, and rhesus macaque. Small spacers (<5 kb) excluded from analysis. Results (A) for all sequence and (B) after masking protein-coding genes (gene body plus 1 kb upstream). *P < 0.05, ns = not significant, Mann–Whitney *U*.

Previous studies have proposed that high rates of gene conversion in sex chromosome palindromes could lead to elevated GC content in palindrome arms (Caceres *et al.* 2007; Hallast *et al.* 2013). We calculated GC content for primate X-chromosome palindrome arms relative to flanking sequence, and found significantly higher median GC content in palindrome arms than in flanking sequence across all three species: 46.3% *vs* 41.2% (human), 46.3% *vs* 40.9% (chimpanzee), and 45.2% *vs* 41.0% (rhesus macaque) (*P* < 0.05 for all three species, Mann–Whitney *U*) (Figure 1A). The GC content of flanking sequences is slightly elevated compared to the overall GC content of the human X chromosome (39.5%), while the GC content of palindrome arms is markedly higher. The trend of elevated GC content in palindrome arms was highly consistent across different palindromes, with at least eleven out of 12 palindromes having significantly higher GC content in palindrome arms than flanking sequence within each species (*P* < 1 × 10^−6^ for each significant palindrome, chi-square test with Yates correction, Supplementary Table S1). Given that ten out of 12 conserved primate X-chromosome palindrome arms contain one or more protein-coding genes (Jackson *et al.* 2021), which tend to be GC-rich, we considered the possibility that elevated GC content in primate X-chromosome palindrome arms results from an enrichment of protein-coding genes. However, the difference between GC content in palindrome arms and flanking sequence remained significant after masking protein-coding genes plus their promoters (defined as 1 kb upstream): 44.1% *vs* 40.1% (human), 44.2% *vs* 40.1% (chimpanzee), and 44.1% *vs* 40.5% (rhesus macaque) (*P* < 0.05 for all three species, Mann–Whitney *U*) (Figure 1B). We conclude that high gene conversion rates in primate X-chromosome palindrome arms are associated with elevated GC content, consistent with the hypothesis that frequent gene conversion causes an increase in GC content over time.

Previous studies of molecular evolution in sex chromosome palindromes have used two different genomic regions as controls for comparison to palindrome arms: Flanking sequence (Caceres *et al.* 2007; Swanepoel *et al.* 2020), or the unique sequence that separates palindrome arms, called the spacer (Rozen *et al.* 2003; Geraldes *et al.* 2010; Hallast *et al.* 2013). Given that both spacers and flanking sequence comprise unique sequence, their GC content might be expected to be similar. However, we found that the GC content of spacers occupied an intermediate range between arms and flanking sequence, and did not differ significantly from palindrome arms (Figure 1, A and B). This finding may be explained by a recent observation that palindrome spacers are structurally unstable on the timescale of primate evolution: For 7/12 palindromes conserved between human and rhesus macaque, spacer sequence could not be aligned between species, and for five palindromes, part of the spacer from one species corresponded to arm sequence in the other (Jackson *et al.* 2021). We suggest that palindrome spacers display an intermediate level of GC content because some spacers spent part of their evolutionary history in the palindrome arm, where they were subject to higher levels of gene conversion. There were also examples of X-chromosome palindromes for which part of the arm in one species corresponded to flanking sequence in another (*e.g.*, P9 in human and rhesus macaque, Jackson *et al.* 2021); this phenomenon may explain why flanking sequence has slightly higher GC content than the X-chromosome average, as noted above.

### Nucleotide replacement patterns in human and chimpanzee X-chromosome palindrome arms demonstrate that GC content has increased in the past 7 million years

**Figure 2:**
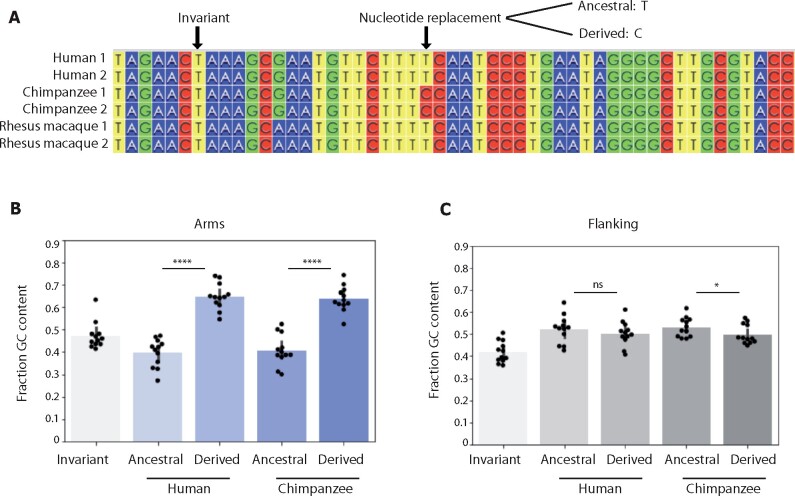
Nucleotide replacements in human and chimpanzee X-chromosome palindrome arms in the past 7 million years have been GC-biased. (A) Identification of nucleotide replacements from six-way arm alignments from palindromes conserved between human, chimpanzee, and rhesus macaque. Invariant sites are identical in human, chimpanzee, and rhesus macaque. Alignments generated with ClustalW and visualized using Wasabi (Veidenberg et al. 2016). (B, C) Fraction GC content for ancestral *vs* derived bases. ****P < 0.0001, *P < 0.05, Mann–Whitney *U*.

We next looked for evidence of GC-biased gene conversion based on nucleotide replacement patterns in palindrome arms. For each conserved X-chromosome palindrome, we generated a six-way alignment using both palindrome arms from human, chimpanzee, and rhesus macaque. We then identified nucleotide replacements that occurred in the human lineage by searching for sites with the same base in both human arms (*e.g.*, G/G) and a different base in rhesus macaque and chimpanzee arms (*e.g.*, A/A in both species) (Figure 2A). Such fixed differences can be inferred to have arisen through a substitution in the human lineage, followed by gene conversion between human arms (Hallast *et al.* 2013, Supplementary Note S1). We compared the base composition of the ancestral base at each site of inferred gene conversion to the derived base. If gene conversion is GC-biased, then derived bases should have a higher GC content than ancestral bases. Indeed, we found that the median GC content of derived bases was 64.5%, compared to 41.5% for ancestral bases (*P* < 0.0001, Mann–Whitney *U*) (Figure 2B). We repeated the same analysis for nucleotide replacements in the chimpanzee lineage, with similar results (62.7% *vs* 39.4%, *P* < 0.0001, Mann–Whitney *U*) (Figure 2B). In contrast, a comparable analysis examining the GC content of ancestral *vs* derived sequence for flanking sequence, using three-way alignments between species, revealed little or no significant difference in base-pair composition (Figure 2C). We conclude that GC-biased gene conversion in human and chimpanzee palindrome arms over the past 7 million years has skewed nucleotide replacement patterns, resulting in derived bases being more than one-and-a-half times more GC rich than the ancestral bases that they replaced. Finally, we used nucleotide replacement patterns to calculate the equilibrium GC content (GC*), which represents the GC content that would be reached at equilibrium if substitution patterns remained constant over time. We found that GC* for primate X palindrome arms is 60.9%, compared to only 39.9% for flanking sequence. While the GC content of primate X palindrome arms has increased over the past 7 million years, we conclude that it is still nearly 15% below its equilibrium value, and thus likely to continue increasing over time.

### Simulations of palindrome gene conversion are consistent with GC bias of about 0.7

**Figure 3:**
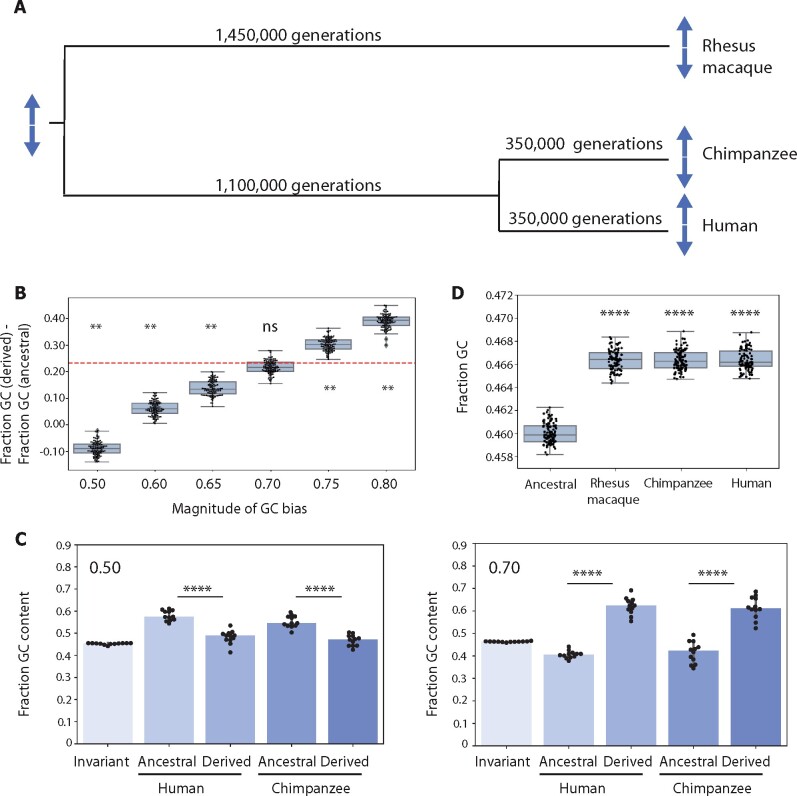
Simulating palindrome evolution with different degrees of GC bias. (A) Schematic of simulations. (B) Simulated differences between GC content of ancestral and derived bases for six different magnitudes of GC bias. Each dot (*n* = 100 for each magnitude of GC bias) represents the median difference for a set of 12 simulated palindromes. Dashed red line represents true value observed in Figure 2B. **P < 0.01, ns = not significant, bootstrapping. (C) Fraction GC content for ancestral *vs* derived bases in simulated palindromes. Results shown for one representative set of 12 palindromes from simulations in Figure 3B. Upper left corner: Magnitude of GC bias. ****P < 0.0001, *P < 0.05, Mann–Whitney *U*. (D) Fraction GC content for simulated palindrome arms and ancestral sequence. Magnitude of GC bias = 0.70. Each dot (*n* = 100 for each category) represents median GC content for a set of 12 simulated palindromes. ****P < 0.0001, *P < 0.05, Mann–Whitney *U*.

Our interpretation of the results shown in Figure 2B assumes that all nucleotide replacements result from the same series of evolutionary events, *i.e.*, a substitution followed by gene conversion. Although we consider this the most parsimonious explanation for fixed differences found in a single species, other explanations cannot be excluded (see Supplementary Note S1). We therefore devised a series of Markov chain Monte Carlo (MCMC) simulations to model palindrome evolution under different magnitudes of GC-biased gene conversion. These simulations allowed us to examine the expected behaviors of palindrome evolution within reasonable parameters for substitution rate, neutral substitution patterns, gene conversion rate, and the magnitude of GC bias, without requiring assumptions about the specific evolutionary trajectory of each site. Our simulations were designed to achieve three objectives: (1) determine the likelihood of observing the pattern of nucleotide replacements shown in Figure 2B in the absence of GC-biased gene conversion, (2) find the magnitude of GC-biased gene conversion most consistent with our results in Figure 2B, and (3) determine what fraction of the elevated GC content seen in primate X-chromosome palindrome arms relative to flanking sequence can be attributed to GC-biased gene conversion. While the simulations shown in Figure 3 were run using identical evolutionary parameters except for the magnitude of GC bias, the effects of altering other parameters are explored in Supplementary Notes S2 and S3; none of these parameter modifications altered the major conclusions of these analyses.

Our simulations model the evolution of a palindrome that was present in the common ancestor of human, chimpanzee, and rhesus macaque, and maintained in all three lineages over 29 million years until the present (see Materials and methods). In brief, for each iteration, we initialized an ancestral palindrome conforming to the median characteristics of 12 conserved primate X palindromes, including arm length, total GC content, and arm-to-arm identity. We then subjected the ancestral palindrome to rounds of nucleotide substitution followed by gene conversion, with each round representing one generation (Figure 3A). We determined neutral substitution patterns based on alignments of 3.8 Mb gene-masked flanking sequence; our observed pattern showed a strong preference for transitions over transversions, as well as a preference for GC→AT substitutions over AT→GC substitutions, consistent with previous reports (Petrov and Hartl 1999; Zhang and Gerstein 2003; Duret and Arndt 2008; see Materials and methods). We included two branching events to account for the divergence of each lineage, resulting in three evolved palindromes representing those present today in human, chimpanzee, and rhesus macaque. Each simulation described below represents one hundred trials, each simulating 12 independent palindromes, representative of the 12 palindromes described in Figures 1 and 2.

We first used our simulations to determine the likelihood of observing a median difference in GC content between ancestral bases and derived bases as large as that observed in Figure 2B in the absence of GC-biased gene conversion (GC bias = 0.50). For simplicity, we report only the results of evolved human palindromes, given that the palindromes designated as “human” and “chimpanzee” underwent equivalent evolutionary trajectories in our simulations. Out of 100 simulations run without GC-biased gene conversion, we never observed a median difference in GC content between ancestral and derived bases as large as the true median difference of ∼23% in primate X-chromosome palindromes (Figures 2B and 3, B and C). Indeed, all observed differences were less than zero, demonstrating that in the absence of GC bias, ancestral bases are expected to be more GC-rich than derived bases, reflecting the higher rate of GC→AT substitutions *vs* AT→GC substitutions (Figure 3, B and C). We conclude that our observed pattern of nucleotide replacements in Figure 2B is unlikely (*P* < 0.01, bootstrapping) in the absence of GC-biased gene conversion.

We next asked what magnitude of GC-biased gene conversion could best explain our observed results in Figure 2B. We repeated our simulations using magnitudes of GC bias ranging from 0.60 to 0.80. Simulations using GC bias of 0.75 and 0.80 both produced median differences in GC content between ancestral and derived bases that were significantly larger than our observed value of 23% (31.8% and 39.0%, respectively, *P* < 0.01 for both), while simulations using GC bias of 0.60 and 0.65 produced values that were significantly smaller (6.8% and 13.8%, respectively, *P* < 0.01 and *P* < 0.01) (Figure 3C). We found that an intermediate value of 0.70 produced results highly consistent with our observations, with a median difference in GC content between ancestral and derived bases of 21.8% (ns, Figure 3C). We conclude that our results in Figure 2B are best explained by a magnitude of GC bias of approximately 0.70, consistent with previous estimates derived from analyses of human meioses (Williams *et al.* 2015; Halldorsson *et al.* 2016).

Finally, we used our simulations to explore the increase in GC content in palindrome arms that would be produced by GC-biased gene conversion of our inferred magnitude, 0.70, over 29 million years of evolution. In particular, we asked what fraction of the difference in GC content observed between palindrome arms and flanking sequence—ranging from 3.6% in rhesus macaque to 4.1% in chimpanzee, after masking protein-coding genes (Figure 1)—could be explained by GC-biased gene conversion over this time scale. We compared the GC content in simulated human, chimpanzee, and rhesus macaque arms to the GC content of the ancestral palindrome. While the three evolved palindromes had significantly higher GC content than the ancestral palindrome, it was by a median magnitude of 0.68%, explaining at most 19% of our observed difference from primate X-chromosome palindromes (Figure 3D). We considered the possibility that GC content might increase by a greater magnitude if the initial GC content of the ancestral palindrome were lower, given that the effects of GC-biased gene conversion tend to be larger when GC content is farther from its equilibrium (Bolívar *et al.* 2016). However, when we repeated our simulations using a magnitude of GC bias of 0.70 and an initial GC content of 0.40, we found only a modest change in the increase in GC content: GC content increased by 0.89% (Supplementary Figure S1). Both values for initial GC content (0.40 and 0.46) are far from the equilibrium value of 0.61 reported above, which may explain the small effect size. While GC-biased gene conversion leads to a significant increase in GC content over time, our results suggest that an increase of the magnitude we observed in Figure 1 is unlikely to have occurred since the divergence of human, chimpanzee, and rhesus macaque. Indeed, the total divergence separating human and rhesus macaque is only around 5.5%, which is expected to be split roughly equally between the two lineages; we therefore infer that neither species should accumulate more than a 2.75% increase in GC content relative to the ancestral palindrome, in the unlikely scenario that every substitution changed an AT base to a GC base. We conclude that either primate X-chromosome palindromes are considerably older than 29 million years, or that other factors contribute to the difference (see Discussion).

## Discussion

GC-biased gene conversion is a powerful force that shapes nucleotide composition across mammalian genomes (Galtier *et al.* 2001; Marais 2003; Duret and Galtier 2009). Previous reports have estimated the magnitude of GC bias in humans to be around 68%, based on the detection of autosomal gene conversion events from three-generation pedigrees (Williams *et al.* 2015; Halldorsson *et al.* 2016). Here, we inferred a magnitude of GC bias of around 70% in a unique system of 12 large palindromes conserved on the X chromosome, using a comparative genomic approach combined with evolutionary simulations. The concordance between our results and those of previous studies, including investigations of GC-biased gene conversion in human Y chromosome palindromes (Hallast *et al.* 2013; Skov *et al.* 2017), suggests that the magnitude of GC bias in humans is relatively constant across diverse genomic contexts. From this, we further infer that regional differences in the effects of GC-biased gene conversion—such as the GC-skewed nucleotide replacements that we detect in primate X-chromosome palindrome arms—stem from regional differences in the rate of gene conversion, rather than in the strength of GC bias.

Previous work has shown that high rates of gene conversion are associated with elevated GC content in ribosomal arrays (Galtier *et al.* 2001), multi-copy histone gene families (Galtier 2003), and human segmental duplications genome-wide (Zhang *et al.* 2005). However, few previous studies have examined the GC content of sex chromosome palindrome arms. One human X-chromosome palindrome with putative orthologues in other mammals was found to have higher GC content in palindrome arms compared to flanking sequence in all sixteen species studied (Caceres *et al.* 2007). Results based on six human Y chromosome palindromes were mixed, with two palindromes showing significantly higher GC content in arms than in spacer, and the other four palindromes showing no significant difference (Hallast *et al.* 2013). The selection of the spacer for comparison may have reduced the significance of the latter findings, given that we found significant results only from comparing GC content between palindrome arms and flanking sequence. In general, we propose that flanking sequence represents a stronger comparison than spacers for molecular analyses of palindrome evolution, due to the fact some X-chromosome palindrome spacers have mixed evolutionary histories that may include time spent within the palindrome arm (Jackson *et al.* 2021).

Although we found that GC content in primate X-chromosome palindromes is robustly elevated in palindrome arms *vs* flanking sequence, simulations show that less than 20% of this increase can be attributed to GC-biased gene conversion since the divergence of the human and rhesus macaque lineages. One possible explanation is that palindromes arose much earlier in primate or mammalian evolution, resulting in additional time to accumulate GC content. However, given the order-of-magnitude difference between our observed results and simulations, we consider under-estimation of palindrome age unlikely to explain the entire discrepancy. We instead propose two mutually compatible possibilities: that GC-rich sequence is more susceptible to palindrome formation, and/or that GC-rich palindromes are more likely to survive over long evolutionary timescales. Both possibilities are bolstered by the fact that although high rates of recombination can elevate GC content over time (Montoya-Burgos *et al.* 2003; Li *et al.* 2016), elevated GC content can also increase local rates of recombination (Petes and Merker 2002; Kiktev *et al.* 2018). Given that palindrome formation is believed to require two recombination events (Kuroda-Kawaguchi *et al.* 2001), recombinogenic GC-rich sequence may be more likely than AT-rich sequence to form palindromes. Palindromes with high GC content may also have a survival advantage over palindromes with lower GC content, given that high rates of recombination are required to prevent arms from diverging over time. We speculate that both factors—an increased tendency for GC-rich sequence to form and maintain palindromes, combined with further gains in GC content over time from GC-biased gene conversion—contribute to the remarkably GC-rich palindromes we observe in X-chromosome palindromes from human, chimpanzee, and rhesus macaque.

## Data availability

BAC sequences used for this study are available from GenBank (https://www.ncbi.nlm.nih.gov/) under accession numbers listed in Supplementary Table S2. The authors affirm that all other data necessary for confirming the conclusions of the article are present within the article, figures and tables. Code used to generate the simulated data can be found at https://github.com/ejackson054/GC-biased-gene-conversion.

Supplementary material is available at *G3* online.
